# Repurposing a machine QA platform for medical physics residency management

**DOI:** 10.1002/acm2.70745

**Published:** 2026-08-13

**Authors:** Darren Zuro, Michael Reilly, Jessica Salas, Jiayi Liu, Maryam Shirmohammad, Xinyi Li, Theodore Geoghegan, Peter Maxim, Mengying Shi

**Affiliations:** ^1^ Department of Radiation Oncology University of California Irvine Orange California USA

**Keywords:** CAMPEP, competency tracking, machine QA software, medical physics residency, residency management

## Abstract

**Background:**

Medical physics residency programs require reliable systems for organizing rotation files, documenting educational activities, recording competency sign‐offs, and monitoring resident progression. Current residency standards from the Commission on Accreditation of Medical Physics Educational Programs (CAMPEP) require clearly defined training schedules, rotation objectives, resident progress evaluation, and regular documentation of trainee development. Many programs continue to rely on static documents, spreadsheets, or multiple disconnected platforms, which may create version‐control problems, increase administrative burden, and reduce transparency. Prior reports have described dedicated educational‐management software (Typhon Group and MedHub) for this purpose, but these require separate licensing and onboarding. RadMachine (Radformation, Inc., New York, NY) is widely used in radiation oncology departments for machine quality assurance (QA) management; repurposing such an existing QA platform for residency management has not been previously described.

**Purpose:**

To describe the repurposing of RadMachine as a platform for medical physics residency rotation organization, activity tracking, competency sign‐offs, and compliance with residency documentation needs.

**Methods:**

A residency management framework was developed within the RadMachine platform. Each resident was configured as a virtual device. Test lists were created and assigned to each resident to represent clinical rotations, an operation sign‐off list, a proficiency checklist, and a comprehensive examination. Individual tests represented, but were not limited to, learning objectives, practical tasks, reading topics, or sign‐off requirements. Completion status was recorded in various ways, including binary completion states, supervisor sign‐offs, detailed comments, or report upload. The prior Microsoft Word–based workflows were qualitatively compared with the proposed RadMachine‐based framework.

**Results:**

The RadMachine‐based framework transformed static training documents into dynamic, trackable test lists. Compared with the prior workflow, the new framework improved version control, reduced administrative effort, simplified updates, allowed transparency, and ensured access to the most current curriculum. Residents could view completed and pending requirements in real time, while faculty could rapidly review trainee progress and outstanding competencies.

**Conclusions:**

Implementing the machine QA software RadMachine for residency education provides a practical, scalable, and low‐overhead solution for rotation management and competency tracking. Because it reuses cloud‐based QA software already licensed and deployed in the department for routine clinical QA—that is, existing departmental infrastructure—the approach requires no additional procurement, licensing, or information‐technology onboarding. This approach is particularly useful for residency programs seeking efficient alternatives to document‐based systems and offers strong potential for future expansion.

## 1 INTRODUCTION

Medical physics residency programs are responsible for preparing trainees for safe and independent clinical practice through structured supervised education. In addition to direct clinical training, programs must maintain organized documentation of curricular content, resident progress, competencies, evaluations, and professional development. These needs are particularly relevant for programs accredited by the Commission on Accreditation of Medical Physics Educational Programs (CAMPEP).

The 2024 CAMPEP Standards for Accreditation of Residency Educational Programs in Medical Physics^1^ specify that residency programs must maintain clearly defined training schedules, clinical rotation objectives, didactic educational expectations, documentation of resident progress evaluation, reading resources, and records of resident activities journals examined regularly by the program director. The standards also emphasize consistently applied metrics for evaluating resident progress and performance. The executive summary of AAPM Report No. 249 outlined the documentation infrastructure expected of accredited clinical medical physics residency training programs;^2^ these essentials were recently revised and expanded in AAPM Task Group Report No. 249.B (2025).^3^

Many residency programs continue to rely on conventional administrative tools such as text documents, spreadsheets, shared folders, or combinations of multiple platforms. While functional, these systems may become increasingly difficult to maintain as programs expand and persist over time. Common challenges include confusion over different versions of program documents, a high administrative workload, scattered records of resident progress, and difficulty obtaining a clear overview of resident progress.

Prior published work in the Journal of Applied Clinical Medical Physics has explored software‐based approaches to these challenges. Zacarias and Mills reviewed a commercially available allied‐health educational‐management platform (Typhon Group) for a CAMPEP‐accredited therapy physics residency program, demonstrating that such software can organize didactic courses, competency reporting by topic and category, time logs, student case reporting, and multi‐site approval of competencies.^4^ Schubert and Miften adapted a commercial web‐based medical residency management system (MedHub) to a medical physics residency program and reported 21 months of program‐level outcomes including centralized document distribution, conference‐attendance records, clinical‐procedure logging across 37 procedure types, automated progress reports, and an integrated electronic evaluation system.^5^ While these dedicated educational platforms are effective, they typically require additional licensing, procurement, information‐technology (IT) integration, and vendor onboarding that may not be practical for smaller programs or programs with limited administrative support. In parallel, open‐source quality assurance (QA) platforms such as QATrack+ have demonstrated that task‐list, test, and sign‐off abstractions generalize well across clinical workflows,^6^ suggesting that a QA software already deployed in a department can be reused for educational documentation at low incremental cost.

At our department, residency rotation curricula and program documents were historically maintained as separate Microsoft Word and Excel files (Microsoft Corp., Redmond, WA) stored within Microsoft Teams and on a departmental shared drive. This approach required frequent manual editing, uploading of revised files, and ongoing folder organization. As the program grew, maintaining this framework became increasingly time‐consuming.

RadMachine (Radformation, Inc., New York, NY) is a web‐based Software‐as‐a‐Service (SaaS) platform designed to support QA in radiation oncology. It provides tools for tracking QA activities, managing equipment and schedules, and organizing documentation within a centralized interface, while enabling standardized data collection, automated task assignment, and QA results tracking. Although developed for physics QA operations, its structured framework—based on task assignment, completion tracking, historical records, and user sign‐off—aligns well with the needs of residency education. In contrast to the prior reports that adopted dedicated educational‐management platforms,^2^, ^3^ the present work demonstrates that a QA software already installed in the radiation oncology department can be repurposed for residency administration without additional procurement, licensing, or vendor onboarding. In this work, we describe the qualitative operational experience and implementation of RadMachine as a centralized platform for medical physics residency management.

## 2 MATERIALS AND METHODS

### 2.1 Residency program structure

Our medical physics residency is a CAMPEP‐accredited program in radiation oncology medical physics. The curriculum is organized into sequential clinical rotations—introduction/orientation, external‐beam treatment planning, machine QA, imaging, brachytherapy, special procedures, clinical projects, and full clinical participation—each with defined objectives, required reading, and competency sign‐offs. In addition to the rotations, residents complete (1) a linear‐accelerator operation sign‐off during the initial orientation, (2) a longitudinal proficiency list of required clinical activities maintained throughout the residency, and (3) a two‐week comprehensive examination, combining a written examination with supervised clinical tasks, prior to graduation. Resident progress is reviewed in regular meetings between the resident and the program director, consistent with CAMPEP requirements. Two residents are currently enrolled. Because residency structures differ between programs, the framework below is described in terms of these generic components—rotations, sign‐off lists, a longitudinal proficiency list, and a comprehensive examination—so that it can be adapted to other program designs.

### 2.2 Department RadMachine setup

**FIGURE 1:**
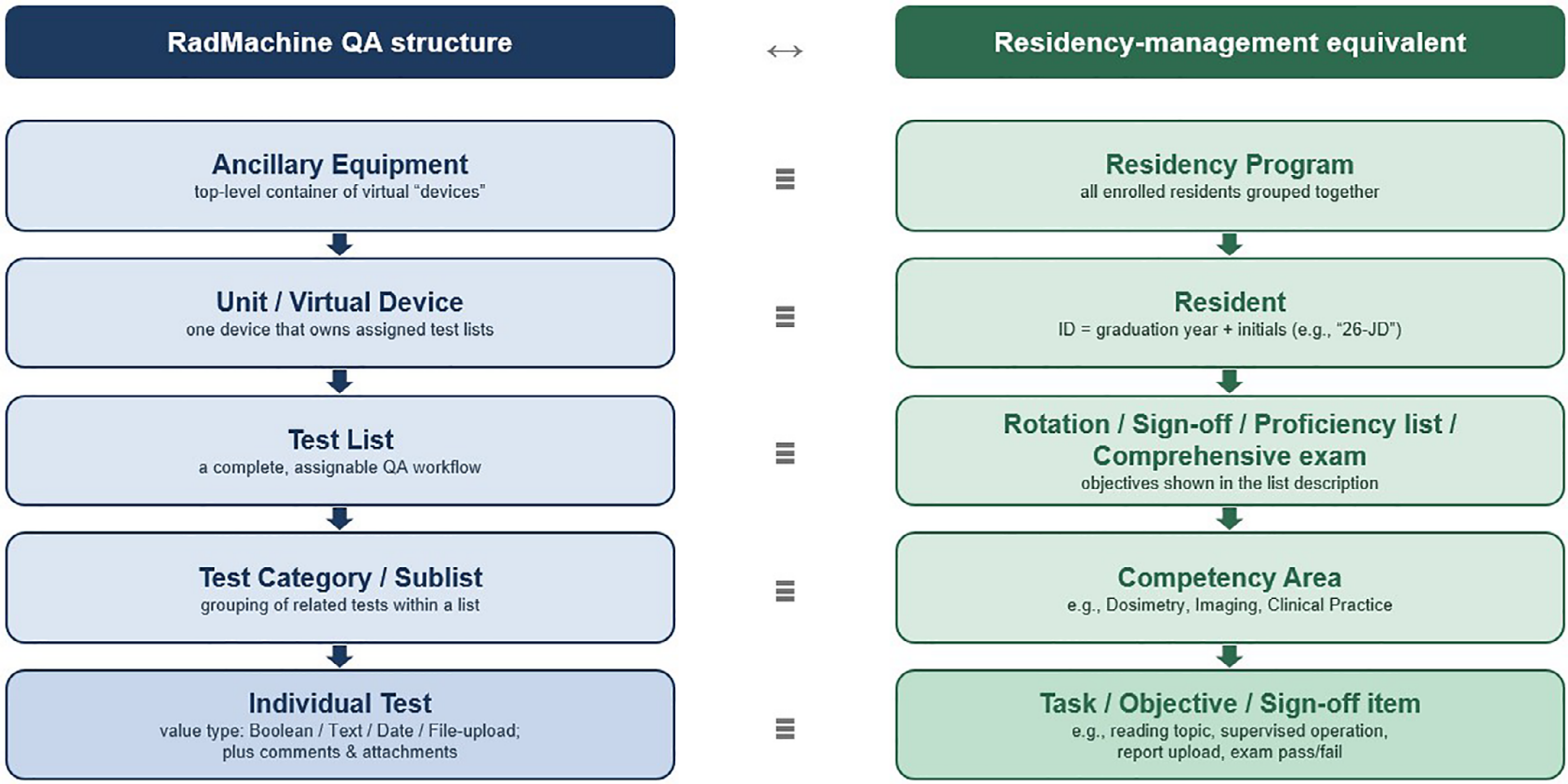
Mapping of the native RadMachine quality‐assurance (QA) hierarchy to the residency‐management framework. Each level of the QA data model (ancillary equipment, unit/virtual device, test list, test category, and individual test) is repurposed to a corresponding level of residency management (program, resident, rotation or checklist, competency area, and individual task or sign‐off item). Arrows denote containment (“is composed of”).

RadMachine was originally developed to support routine machine QA workflows and can interface with multiple radiation therapy delivery platforms and treatment planning systems from different vendors. QA configuration within RadMachine follows a hierarchical, pyramid‐based structure (Figure 1). The foundational element is an individual test, the smallest unit of data entry; each test is assigned a value type that determines how its result is recorded—for example, Boolean (pass/fail or yes/no), numeric, text, date, or file upload. Related tests are grouped into a test list, which defines a complete, assignable workflow analogous to a single checklist or procedure; the objectives or instructions for a list are shown in its description field. A test list with a certain frequency is assigned to a unit—the equipment or entity that “performs” the list. A single unit may have several test lists associated with it, and, conversely, a given test list may be assigned to multiple units, enabling standardized protocols to be reused across similar systems. In routine clinical use, units correspond to treatment machines or QA devices; in the present application, each unit is a resident (see RadMachine Framework, below). Tests within a list may be further organized into named categories or sublists for clarity. Figure 1 summarizes how each level of this native QA hierarchy is repurposed for residency management.

Initial system configuration requires a dedicated full‐time effort to establish test lists and unit assignments. However, once test lists are created, the automated ingestion of test data and built‐in analysis tools significantly reduce ongoing operational effort. An additional key feature of RadMachine is comprehensive audit and log tracking. Any creation or modification of tests, test lists, or unit assignments is automatically recorded and retained for the lifetime of the object, with time‐stamped version tracking to support traceability and regulatory compliance.

### 2.3 Old framework

Commonly used documents for program leadership, mentors, and trainees include rotation curricula, machine operation checklists, comprehensive examination requirements, and resident proficiency checklists.

Before implementation, residency curricula were maintained through standalone Word documents stored in Microsoft Teams folders. Each rotation typically required a separate file describing objectives, required reading, competencies, and sign‐off expectations. Revisions required manual editing followed by redistribution or replacement of stored files. Over time, version control became increasingly challenging when multiple revisions existed or older copies remained accessible. Additional administrative effort was required to organize folders, rename files, and communicate updates to residents and mentors. Tracking resident completion of curricular topics and supervised sign‐offs was not integrated into this model. Therefore, the progress review required separate communication or additional records outside the curriculum files.

At our department, physics residents were required to complete sign‐off of basic linear accelerator operation during the initial orientation rotation. In addition, residents were expected to fulfill designated tasks from a program proficiency list. Prior to graduation, residents are required to complete a mandatory two‐week comprehensive examination that includes multiple clinical activities as well as a written exam. These processes were managed through separate Word‐based documentation and were subject to many of the same limitations as the rotation curricula, including scattered records, administrative burden, and version‐control challenges, highlighting the need for a more practical management solution.

### 2.4 RadMachine framework

**FIGURE 2:**
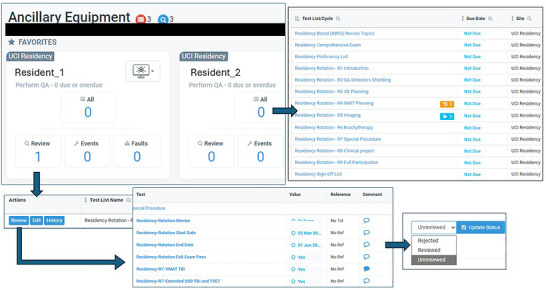
RadMachine home page showing residents represented as virtual devices. Each enrolled resident appears as a card under the “Ancillary Equipment” tab (resident identities are de‐identified here as “Resident_1” and “Resident_2”). The dashboard summarizes, for each resident, the number of test lists due or overdue and provides “Review,” “Events,” and “Faults” counters. The expanded panel (right) lists all rotation, sign‐off, proficiency, comprehensive‐examination, and board‐review test lists assigned to a resident, together with their due dates and progress status.

RadMachine was adapted for this use by applying its built‐in QA structure to an educational setting. Each resident was configured into the system as an individual virtual equipment with an identifier consisting of their graduation year and name initials. This allowed resident‐specific assignment of test lists and sign‐offs, review of completion status, and longitudinal tracking using existing software functionality. All residents are listed under the “Ancillary Equipment” section on the RadMachine home page. Multiple test lists are assigned to each resident. See Figure 2 for an example. At present, two residents are enrolled in our medical physics residency program and are available within the RadMachine system. The test lists assigned to each resident can be viewed under the “All” tab.

**FIGURE 3:**
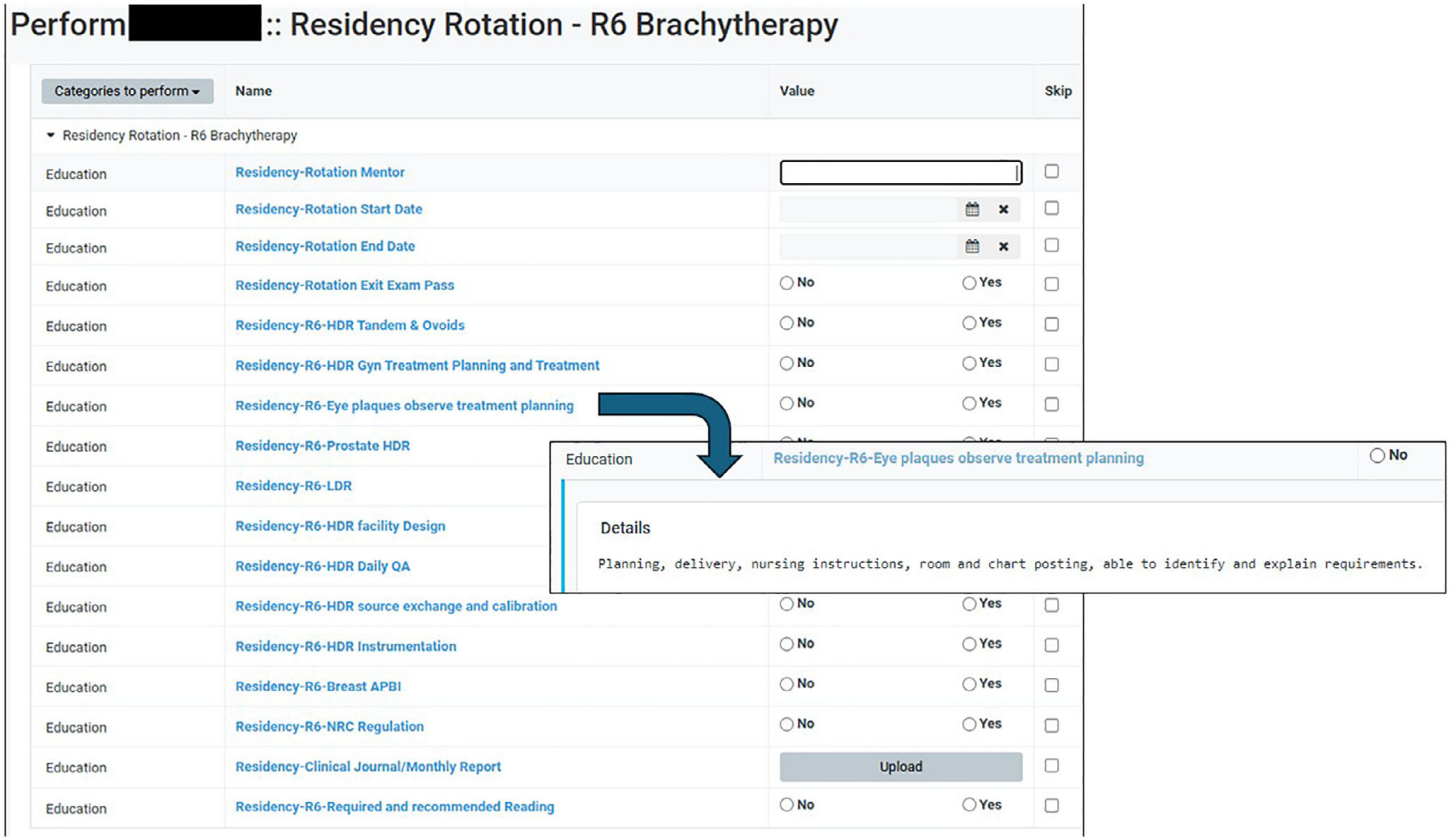
Example of a clinical‐rotation test list in RadMachine. Rotation objectives are shown in the list description; individual tests use the value type appropriate to their purpose (text for the assigned mentor, dates for rotation start and end, Boolean checkboxes for reading topics and study objectives, and file upload for the resident clinical journal), with per‐item instructions and references displayed beneath each test.

Test lists were created to represent clinical rotation curricula. Within each rotation test list, the educational objectives were displayed in the description section for review by both the resident and assigned mentor. Each list contained a series of individual tests configured with different data‐entry formats according to the intended purpose. A text‐type value was assigned to the item identifying the rotation mentor. Date‐type values were assigned to the rotation start and end dates. Boolean‐type values were used for exit examination pass status, completion of individual study topics, and required reading assignments. A file‐upload type value was used for submission of the resident clinical journal. Additional details for each study topic were displayed under the corresponding test item for resident reference and review. Residents were responsible for completing the required test fields throughout the rotation and submitting the finalized record at the end of the rotation for the mentors and program director to review. A sample of the rotation test list is displayed in Figure 3. The resident can partially complete a test list and submit the “QA” results while it is marked as “In Progress,” allowing the list to be edited at a later time. Once the list is fully completed, the resident can submit the results for final review.

As a concrete example of the transformation, consider the external‐beam treatment‐planning rotation (Table 1). Under the old framework, this rotation existed as a single Microsoft Word file in a Teams folder that listed the rotation objectives, a required‐reading list, the competencies to be signed off, and a space for the mentor's name; tracking which items a resident had completed required separate emails or a manually updated copy. Under the new framework, the same rotation is a single RadMachine test list (Figure 3). The objectives appear in the list description, the mentor is captured in a text field, the rotation start and end dates in date fields, each required‐reading item and study topic as its own Boolean test, and the resident's clinical journal as a file‐upload field. The resident updates items as they are completed, saves the list as “In Progress,” and submits it for mentor and program‐director review when finished, so that completion status, supporting documents, and the full revision history all reside in one place.

**TABLE 1:** Worked example of one clinical rotation (external‐beam treatment planning) transformed from the document‐based framework to the RadMachine‐based framework.

Rotation element	Old framework (word / teams)	New framework (RadMachine)
Rotation objectives	Narrative text in a Word file	List description field
Assigned mentor	Typed into the document	Text‐type test
Rotation start / end dates	Typed into the document	Date‐type tests
Required reading	Bulleted list; completion tracked separately	One Boolean test per item, with the reference embedded beneath
Study topics / competencies	Checklist in the document	One Boolean test per topic
Resident clinical journal	Emailed or stored separately	File‐upload test
Progress tracking	Manual (email or updated copy)	Real‐time status; “In Progress” → submitted for review
Version history	Multiple file copies	Automatic time‐stamped audit log

**FIGURE 4:**
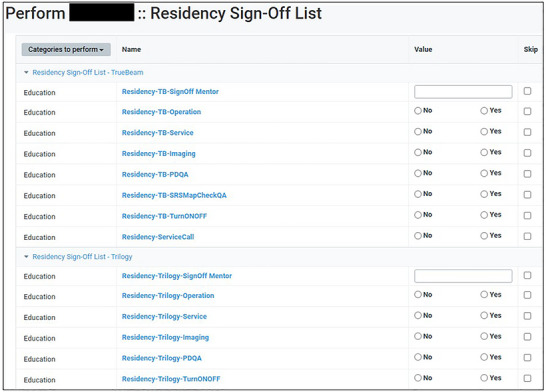
Example of a linear‐accelerator operation sign‐off list in RadMachine. Each operational competency (e.g., treatment‐mode operation, image acquisition, patient‐specific QA delivery, on/off and service‐mode operation) is recorded as a Boolean item that the supervising QMP marks as completed once the resident demonstrates competency; per‐task instructions are embedded beneath each item.

The linear‐accelerator operation sign‐off list was developed for two machine types (Varian TrueBeam and Varian Trilogy), with each containing a defined set of operational tasks that residents were required to demonstrate competently to a Qualified Medical Physicist (QMP). The tasks included operation in treatment mode, image acquisition, delivery of patient‐specific QA plans, powering the machine on and off, operation in service mode, and contacting the field service engineer for service‐related issues. Residents who successfully completed the full sign‐off list were considered eligible to operate the linear accelerator independently. Boolean‐type values were assigned to each operational item to document completion status. Additional details and instructions for each task were embedded under the corresponding test item for resident reference and review. Residents were responsible for performing each required operation under direct supervision of a QMP and completing the associated documentation. The finalized sign‐off record was then submitted for review by the program director. A representative example of this sign‐off list is shown in Figure 4.

**FIGURE 5:**
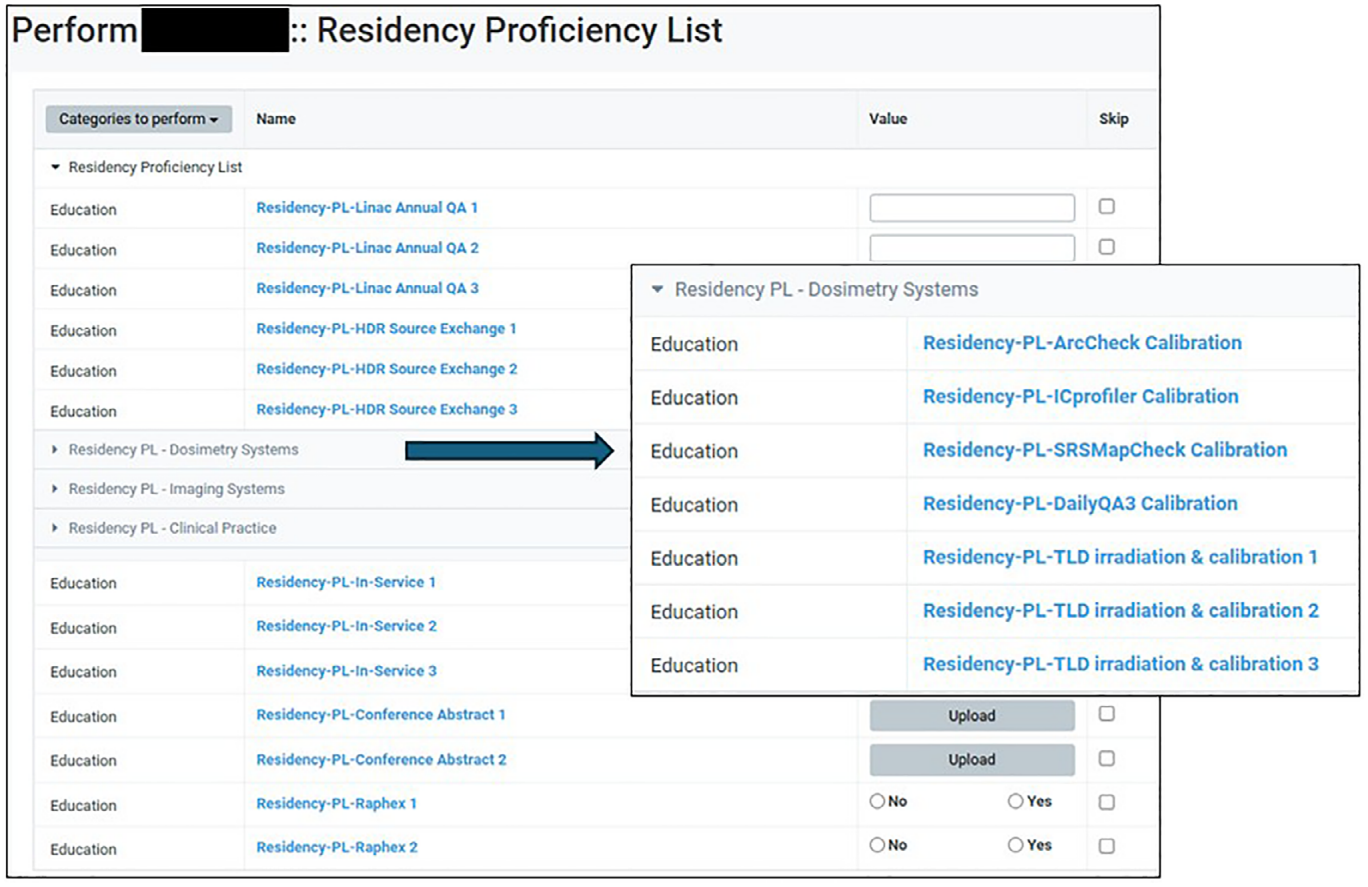
Example of a resident proficiency list in RadMachine. Required clinical activities are grouped into expandable competency categories (e.g., “Dosimetry Systems,” “Imaging Systems,” “Clinical Practice”). Completion is documented with Boolean fields, text or numeric fields (for details such as the supervising QMP, date, and machine identifier), and file‐upload fields (e.g., for conference abstracts and in‐service presentation slides). The supervising QMP signs off each item, and the completed list is submitted to the program director for review prior to graduation.

Each resident is assigned a “Proficiency List” that outlines the required clinical activities and training requirements throughout the residency period. These activities include, but are not limited to, a specified number of annual and monthly machine QA procedures, HDR source exchanges, detector and imaging‐system calibrations, treatment planning exercises, and simulation practice as seen in Figure 5. Both Boolean and text fields are used to document completion. Residents may enter relevant details—such as the supervising QMP, date, and machine ID—either in designated text fields or in the comments section. Where a competency is expected to develop through graded levels of responsibility rather than a single “performed” milestone, this progression can be represented within a single competency by multiple Boolean items (for example, “observed,” “performed under direct supervision,” and “performed independently”) or by a text or numeric field recording the supervision level, allowing progressive responsibility to be documented over time. Residents are also encouraged to deliver in‐service presentations to the department and to participate in conferences. A file‐upload field is used for submitting presentation slides and conference abstracts. In addition, residents are encouraged to complete departmental written mock exams (e.g., using Raphex) and log the completion in RadMachine. The finalized sign‐off record is submitted to the program director for review prior to graduation. An example of this sign‐off list is shown in Figure 5.

Across all list types, the preceptor or program director performs sign‐off through RadMachine's built‐in review workflow. Once a resident submits a completed list, it enters a “Review” queue—reflected in the “Review” counter on each resident's dashboard (Figure 2)—where the reviewer can inspect the entered values and uploaded reports, add free‐text comments, and approve or return the list. This review‐and‐approval step is the mechanism summarized in the “Review” row of Table 2. Because each test also supports free‐text comments, the resident, mentor, and program director can use the interface for asynchronous feedback and communication tied directly to the relevant task. Reference files and documents—such as required‐reading PDFs, procedure instructions, and planning datasets—can be embedded directly within individual tests as attachments, so that residents access supporting material in the same place where they record completion.

**TABLE 2:** Comparison of the old (document‐based) and new (RadMachine‐based) residency‐management frameworks across key administrative dimensions.

Dimension	Old framework	New framework
Platforms	Word, Teams	RadMachine
Rotation curricula	Separate files	Central database
Version control	Manual	Automated
Updates	Replace uploaded files	Live edits
Progress tracking	Manual	Built‐in
Review	Separate process	Integrated
Visibility control	Folder dependent	Configurable
Historical records	Scattered	Built‐in
Administrative burden	High	Lower

**FIGURE 6:**
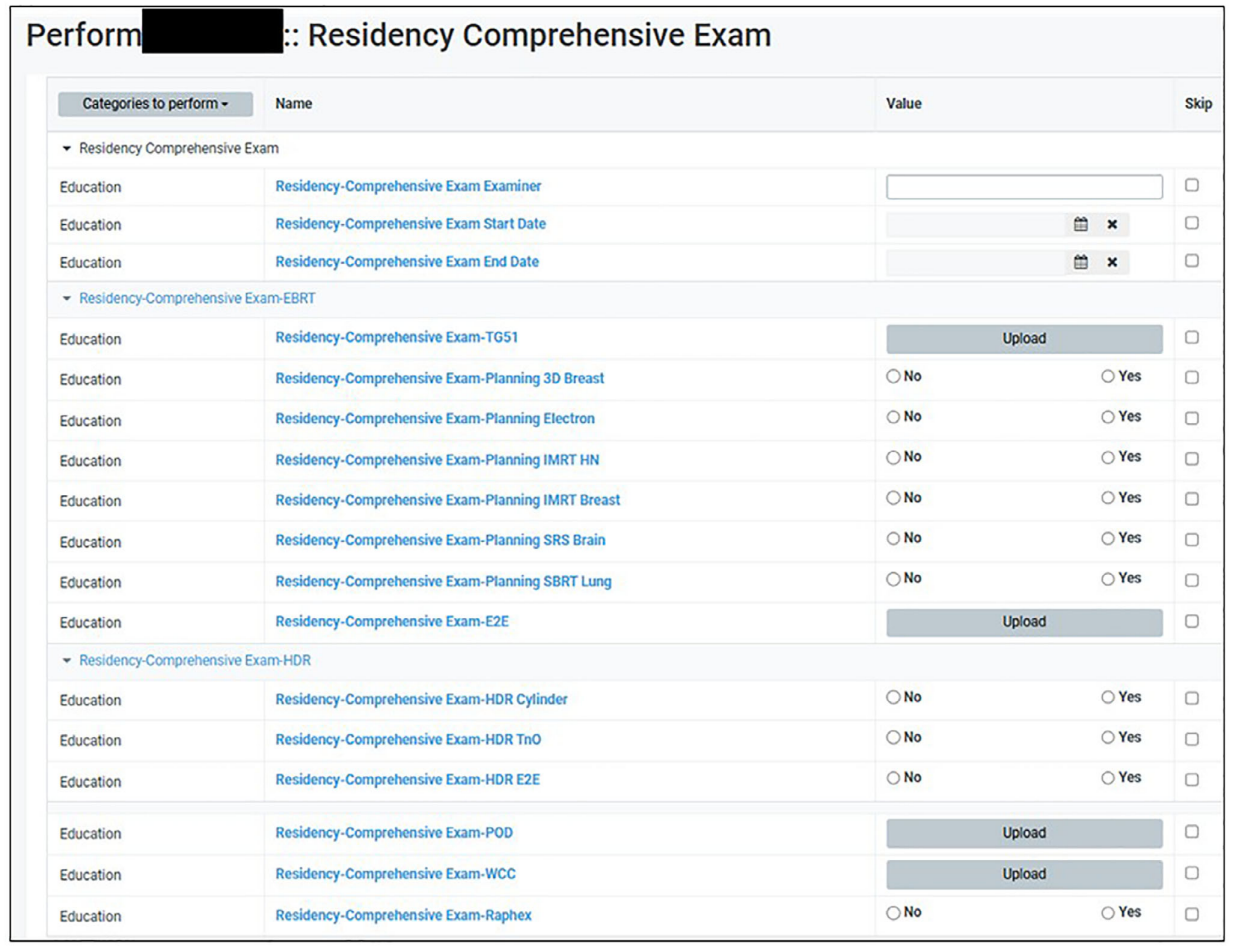
Example of a comprehensive‐examination test list in RadMachine. The two‐week examination is represented as a single test list combining Boolean completion fields and file‐upload fields for calculation and observation reports (e.g., TG‐51 output measurement, end‐to‐end verification, physicist‐of‐the‐day coverage, and weekly chart checks); anonymized DICOM datasets, prescriptions, and planning directives are attached within the relevant tests for the resident to download for planning exercises.

Residents are required to complete a comprehensive examination prior to graduation. At our department, this exam lasts two weeks and includes not only a written exam but also a series of clinical tasks, such as machine output measurement following the TG‐51 protocol, treatment planning practices, external‐beam treatment end‐to‐end (E2E) verification, HDR end‐to‐end demo delivery, physicist‐of‐the‐day (POD) coverage, and weekly chart checks (WCC). All activities are performed under the supervision of a QMP, and results are submitted for the program director's review. A dedicated test list is created in RadMachine and assigned to each resident. For TG‐51, E2E, POD, and WCC tasks, file‐upload fields are used to submit calculation and observation reports, while Boolean fields are used to document completion of other activities. For treatment planning exercises, required materials—such as anonymized DICOM datasets, prescriptions, and planning directives—are provided as attachments within each test. Residents can download and import these into the treatment planning system for practice. The supervising QMP and exam date are recorded at the beginning of the list. A representative example is shown in Figure 6.

Additional test lists—such as new‐member onboarding checklists and board‐exam review topics—can also be created as separate test lists and assigned to residents or physicists. Leveraging the different value types, particularly the “File Upload” option, provides an effective way to collect and organize notes, reports, and other documentation for future reference within the team.

RadMachine supports a variety of test frequencies, including daily, monthly, and annual intervals, which can be assigned to individual test lists, and more customized frequencies can be configured as needed. Due dates can either be pre‐populated when a list is assigned—so that an entire rotation schedule is laid out at the start of the academic year for each resident—or left at an ad hoc frequency that residents self‐manage. At our department, most residency lists use an ad hoc frequency, allowing residents to manage their rotation progress and checklist completion flexibly, while time‐bound items (such as the comprehensive examination) are assigned explicit due dates. Lists are pushed out per resident, either by duplicating a template list at the beginning of each year or by re‐dating an existing assignment, so that scheduling can be handled either up front or incrementally as a resident advances.

At a broader organizational level, such as across a department or training program, RadMachine test lists can be used to track personnel certification status and clinical procedure guidelines or standard operating procedures (SOPs). In these cases, visibility settings and editing permissions should be carefully controlled to ensure appropriate access and protect critical information.

### 2.5 Hybrid framework approach

Although RadMachine was adopted as the primary platform for curriculum organization, activity tracking, and competency sign‐offs, not all residency materials were transferred during the implementation. Certain records required additional confidentiality protections or were already supported efficiently in existing institutional systems. Accordingly, a hybrid framework was maintained at our physics residency program. Resident rotation evaluations continued to be recorded in a separate platform to preserve privacy and maintain established evaluation processes. The existing Microsoft Teams channel dedicated to the residency program also remained active. Private channels were used for sensitive materials such as interview records and exit examination questions, whereas public channels continued to store program policies, announcements, and shared reference resources.

## 3 RESULTS

The RadMachine‐based framework converted multiple static residency documents into a centralized and continuously updated dynamic database environment. Study materials no longer required maintenance as separate documents distributed across folders or Teams channels. Educational content could be edited directly within the framework, and authorized users such as program leadership, mentors, and residents immediately viewed the most current version.

A comparison between the old and the new frameworks is listed in Table 2. Compared with the old Word and Teams workflow, version control of residency documents improved greatly. The old process occasionally created uncertainty regarding whether a stored file represented the latest curriculum revision unless carefully labeled. In contrast, the historical edits and completion records on RadMachine remained preserved within the database. Administrative burden was reduced because updates no longer required repeated file editing, renaming, uploading, or reorganizing. New topics or competencies could be added directly into existing test lists, and outdated items could be revised without reconstructing documents. Trainee transparency improved because the residents could directly review completed tasks, pending requirements, and expectations for each rotation in real time. Trainer oversight also became more efficient. Program leadership and mentors could rapidly review completion status, identify outstanding competencies, and prepare for periodic evaluations without requesting separate reviewing files. This functionality was particularly useful for regular meetings between the program director and residents, which are required by CAMPEP standards.

Longitudinal progress could be reviewed at a glance from the resident dashboard (Figure 2), which aggregates the number of due and overdue lists together with the review status for each resident, functioning as a lightweight progress dashboard. At the item level, the per‐test history view preserves a time‐stamped record of every entry and revision for the lifetime of the object, so that a resident's development across a rotation—or across the entire residency—can be reconstructed and audited without assembling separate files.

After successful implementation for rotations, the same architecture was extended to additional program functions such as proficiency checklists, machine operation sign‐offs, and the comprehensive examination. Other functions are also under consideration, such as board‐exam review topics and a new‐employee onboarding checklist. No separate software platform or documents were required for such expansions.

The hybrid model allowed RadMachine to function as the operational core for structured educational workflows while preserving other systems for tasks better suited to confidential file management or communication. This reduced the need to move older materials unnecessarily while preserving workflows that were already working well.

Implementation of RadMachine for residency management required an initial investment in system configuration and familiarity with the platform's test and test‐list structure. Users responsible for setup needed knowledge of test and test‐list creation, value‐type assignment, visibility settings, and list organization. Creation of an individual rotation or checklist typically required approximately 2–4 h, depending on the number and complexity of listed items. At our institution, complete initial configuration of all residency rotations and checklists took approximately 15 h of administrative effort. Once the framework was established, subsequent revisions were more efficient, with most routine edits, additions, or wording updates completed within. Administrative burden was not measured with a formal instrument; it was assessed qualitatively, as the perceived effort and number of manual steps required of program staff, together with the one‐time configuration‐time estimates reported above. No validated workload survey or time‐and‐motion study was performed, and quantifying time savings is identified as future work.

## 4 DISCUSSION

This study describes a practical educational innovation in which an existing machine QA software was successfully repurposed for residency management. The key insight is that many operational needs of residency management are similar to those of a machine QA program. Both require assignment of responsibilities, tracking of completed tasks, documentation of status, controlled visibility, and preservation of historical records. By reinterpreting devices as residents and QA tests as educational tasks, the RadMachine platform became a natural fit for educational administration.

The present approach is complementary to, but distinct from, prior reports of software‐based residency management. Zacarias and Mills^4^ and Schubert and Miften^5^ adapted dedicated educational‐management platforms—Typhon and MedHub, respectively—for CAMPEP medical physics residency programs. These platforms provide native support for narrative evaluations, longitudinal procedure logs, and structured surveys, and they established a useful quantitative benchmark for residency informatics. The contribution of the present work is different: rather than adopting a second vendor platform, we reuse a QA software already present in the radiation oncology department. This avoids additional licensing and IT‐onboarding costs, enables programs with smaller administrative budgets to meet CAMPEP documentation expectations, and provides the secondary benefit of resident familiarity with a platform they will use for routine clinical QA after graduation. Compared with the open‐source alternative QATrack+,^6^ RadMachine offers a commercially supported, audit‐compliant environment at no additional licensing cost for departments that already license it, at the expense of a proprietary dependency.

Although the implementation described here used RadMachine, the underlying concept is not specific to a single vendor. The essential requirement is a QA‐management platform whose data model exposes assignable task lists, configurable item value types (Boolean, numeric, text, date, and file upload), per‐item sign‐off, comments, and an audit trail. Other cloud‐based machine‐QA systems in common use—such as Image Owl TotalQA and Sun Nuclear SunCheck—provide analogous task‐list and review constructs and could, in principle, host an equivalent residency framework, as could the open‐source QATrack+.^6^ Programs should confirm that their chosen platform supports per‐resident (unit‐level) assignment, the value types needed for their checklists, and appropriate visibility and permission controls before migrating educational records. The broader contribution of this work is therefore the mapping of residency‐management needs onto a generic QA data model, rather than any RadMachine‐specific feature.

The relationship to CAMPEP standards is particularly relevant. Residency programs must demonstrate organized curricula, documented objectives, resident activity records, and consistent progress assessment. Document‐based systems can satisfy these requirements, but often through labor‐intensive manual processes. In contrast, the RadMachine framework embedded many of these elements into routine workflow. This may simplify annual program review, self‐study preparation, and retrieval of training documentation, and it aligns with the documentation infrastructure described in the AAPM Report No. 249 executive summary^2^ and its 2025 update, AAPM Task Group Report No. 249.B.^3^

This model may be particularly attractive to residency programs that already use RadMachine clinically, as implementation may require little additional infrastructure. Another advantage for residents is gaining familiarity with the RadMachine system, which they will also use for routine QA. Because test lists are self‐contained objects, an institution's residency lists can be duplicated, exported, or shared as templates; our rotation, sign‐off, proficiency, and comprehensive‐examination templates are available from the authors on reasonable request, which may lower the barrier for programs beginning to build CAMPEP‐aligned documentation. Programs could further adapt the framework for new‐employee onboarding checklists, annual safety checks, board‐exam review topic lists, and employee certification tracking.

Several limitations should be acknowledged. This report reflects a single‐institution operational experience with only two enrolled residents, and outcomes were qualitative rather than based on formal analysis, time‐and‐motion measurement, or validated surveys; the metrics reported here are descriptive and are not directly comparable to the quantitative benchmarks previously established in this journal, which included document upload and download rates, conference attendance, numbers of clinical procedures logged, and evaluation turnaround times over 21 months.^5^ RadMachine was not originally designed as an educational platform, so certain functions—such as narrative evaluations, structured rotation‐evaluation surveys, and advanced learning analytics—may still require supplemental processes; for this reason a hybrid framework is recommended rather than wholesale replacement of existing tools. RadMachine is also a proprietary SaaS, so this specific implementation is not directly portable to programs that do not license it; such programs may obtain similar benefits from open‐source alternatives such as QATrack+^6^ or from the dedicated educational platforms previously described.^2^, ^3^ Future work could include multicenter implementation, resident and faculty satisfaction surveys, and formal estimates of administrative time savings.

An important practical lesson from this implementation is that it does not require complete replacement of all existing residency administrative tools. While RadMachine was particularly effective for structured checklists, progress tracking, and longitudinal documentation, other systems may remain advantageous for confidential evaluations, interview files, or secure examination materials. Future expansion of RadMachine to these applications would likely require customized user‐group permissions and list architecture, advanced visibility controls, and possibly vendor‐supported development. Collaboration with the vendor may represent a valuable next step for institutions interested in broader educational use of the platform.

## 5 CONCLUSION

The repurposing of RadMachine for medical physics residency management provided a practical and efficient alternative to document‐based workflows. By configuring residents as devices and educational assignments as test lists, the new framework enabled centralized curriculum management, progress tracking, sign‐off documentation, and reduced administrative burden. The system also aligned naturally with several documentation expectations described in current CAMPEP residency standards. Rather than replacing all existing tools, RadMachine functioned effectively as part of a hybrid approach. This model may be valuable to other residency programs seeking modern solutions for training organization and accreditation readiness.

## AUTHOR CONTRIBUTIONS

All authors contributed to the development and implementation of the project. Mengying Shi and Darren Zuro drafted the manuscript. All other authors reviewed the manuscript and provided critical feedback and revisions.

## CONFLICT OF INTEREST STATEMENT

The authors declare no conflicts of interest
